# Adsorption of Cationic Dyes on a Magnetic 3D Spongin Scaffold with Nano-Sized Fe_3_O_4_ Cores

**DOI:** 10.3390/md19090512

**Published:** 2021-09-09

**Authors:** Maryam Akbari, Hessam Jafari, Mojtaba Rostami, Gholam Reza Mahdavinia, Ali Sobhani nasab, Dmitry Tsurkan, Iaroslav Petrenko, Mohammad Reza Ganjali, Mehdi Rahimi-Nasrabadi, Hermann Ehrlich

**Affiliations:** 1 Department of Surgery, School of Medicine, Kashan University of Medical Sciences, Kashan 8719657891, Iran; maryam.akbari078@gmail.com; 2 Polymer Research Laboratory, Department of Chemistry, Faculty of Science, University of Maragheh, Maragheh 5518183111, Iran; hessamjafari1996@gmail.com (H.J.); grmnia@maragheh.ac.ir (G.R.M.); 3 School of Chemistry, College of Science, University of Tehran, Tehran 1983969411, Iran; rostami.mojtaba@ut.ac.ir; 4 Social Determinants of Health (SDH) Research Center, Kashan University of Medical Sciences, Kashan 8719657891, Iran; ali.sobhaninasab@gmail.com; 5 Core Research Lab, Kashan University of Medical Sciences, Kashan 8719657891, Iran; 6 Institute for Electronics and Sensor Materials, TU Bergakademie Freiberg, 09599 Freiberg, Germany; tsurkandd@gmail.com (D.T.); iaroslavpetrenko@gmail.com (I.P.); 7 Center of Excellence in Electrochemistry, School of Chemistry, College of Science, University of Tehran, Tehran 1983969411, Iran; ganjali@ut.ac.ir; 8 Biosensor Research Center, Endocrinology and Metabolism Molecular Cellular Sciences Institute, Tehran University of Medical Sciences, Tehran 1983969411, Iran; 9 Chemical Injuries Research Center, Systems Biology and Poisonings Institute, Baqiyatallah University of Medical Sciences, Tehran 1951683759, Iran; 10 Faculty of Pharmacy, Baqiyatallah University of Medical Sciences, Tehran 1951683759, Iran; 11 Center for Advanced Technology, Adam Mickiewicz University, 61614 Poznan, Poland; 12 Centre for Climate Change Research, Toronto, ON M4P 1J4, Canada; 13 Environmental Solutions, ICUBE-University of Toronto Mississauga, Mississauga, ON L5L 1C6, Canada

**Keywords:** marine biopolymers, 3D spongin scaffold, Fe_3_O_4_ nanoparticles, methylene blue, crystal violet, adsorption mechanism

## Abstract

The renewable, proteinaceous, marine biopolymer spongin is yet the focus of modern research. The preparation of a magnetic three-dimensional (3D) spongin scaffold with nano-sized Fe_3_O_4_ cores is reported here for the first time. The formation of this magnetic spongin–Fe_3_O_4_ composite was characterized by X-ray diffraction (XRD), thermogravimetric analysis (TGA), differential thermal analysis (DTA) (TGA-DTA), vibrating sample magnetometer (VSM), Fourier-transform infrared spectroscopy (FTIR), and zeta potential analyses. Field emission scanning electron microscopy (FE-SEM) confirmed the formation of well-dispersed spherical nanoparticles tightly bound to the spongin scaffold. The magnetic spongin–Fe_3_O_4_ composite showed significant removal efficiency for two cationic dyes (i.e., crystal violet (CV) and methylene blue (MB)). Adsorption experiments revealed that the prepared material is a fast, high-capacity (77 mg/g), yet selective adsorbent for MB. This behavior was attributed to the creation of strong electrostatic interactions between the spongin–Fe_3_O_4_ and MB or CV, which was reflected by adsorption mechanism evaluations. The adsorption of MB and CV was found to be a function of pH, with maximum removal performance being observed over a wide pH range (pH = 5.5–11). In this work, we combined Fe_3_O_4_ nanoparticles and spongin scaffold properties into one unique composite, named magnetic spongin scaffold, in our attempt to create a sustainable absorbent for organic wastewater treatment. The appropriative mechanism of adsorption of the cationic dyes on a magnetic 3D spongin scaffold is proposed. Removal of organic dyes and other contaminants is essential to ensure healthy water and prevent various diseases. On the other hand, in many cases, dyes are used as models to demonstrate the adsorption properties of nanostructures. Due to the good absorption properties of magnetic spongin, it can be proposed as a green and uncomplicated adsorbent for the removal of different organic contaminants and, furthermore, as a carrier in drug delivery applications.

## 1. Introduction

Adsorption-based techniques are considered to be among the most efficient technological options for water treatment, especially in industrial organic waste applications. Removal of organic dyes from wastewater using a broad array of adsorbents is still under extensive investigation [1]. Numerous industrially used dyes are highly toxic, resistant to natural degradation, and can seriously damage the environment [2]. Different procedures have been explored to eliminate dye-based pollutants from aqueous solutions, including biochemical, electrochemical, wet chemical, or sonochemical oxidation, adsorption, ozonation, and biodegradation [3]. This study investigated a spongin–Fe_3_O_4_ composite as a possible absorbent of two dyes: methylene blue (MB), and crystal violet (CV).

MB is a cationic dye with high water and alcohol solubility that is commonly used in the textile industry. Inhaling MB can lead to respiratory conditions, and its ingestion can cause burning sensations, nausea, diarrhea, gastritis, abdominal and chest pains, headaches, excessive sweating, and confusion. Therefore, lowering its content—or, preferably, complete removal from water—is crucial [4,5]. CV is also a water-soluble cationic dye commonly used in the textile industry and in printing inks. CV molecules are visible in the water, even at concentrations as low as 1 ppm, and can interfere with the photosynthesis of aquatic plants [6]. Even at 1 ppb, CV has been reported to have toxic and even mutagenic effects on animals and humans [7]. As a result, proper treatment techniques for removing these organic pollutants from wastewater are urgently needed in order to avoid environmental damage.

Magnetic metal oxides, given the presence of active sites on their surfaces, are among the recently investigated candidates for use as adsorbents for wastewater treatment [8]. One of the most promising of these compounds is Fe_3_O_4_ nanoparticles, which provide considerable biocompatibility, low cost, excellent magnetic susceptibility, and potential for functionalization [9]. Even bare Fe_3_O_4_ nanoparticles are known to act as dye absorbents due to their negatively charged surfaces [10]. As part of an environmentally friendly magnetic composite, the implementation of these nanoparticles was our focus in developing a new adsorbent-based water treatment.

In recent years, the synthesis of new composites based on natural compounds for various applications has received much attention [11,12,13]. In this regard, in this work, for the first time, the magnetic composite of spongin was synthesized using a simple method in ambient temperature and pressure, and was used to remove MB and CV dyes. Our goal was to place magnetic Fe_3_O_4_ nanoparticles on the surface of the renewable marine biopolymer spongin [14,15], which resembles the size and shape of 3D skeletal constructs of diverse keratosan (bath) sponges [16,17]. Commonly, spongin has found application in the immobilization of enzymes [18], cultivation of microorganisms, and biomedicine [19,20]. Recently, this naturally pre-structured, mesoporous scaffold has been successfully used for the adsorption of ketamines [21] and fenitrothion [22], as well such dyes as C. I. Natural Red 4 [23], anthocyanin [24], and sodium copper chlorophyllin [25]. It has also been used as support for Iron(III) phthalocyanine as a photocatalyst in the highly efficient removal of halophenols and bisphenols. Immobilization of titanium(IV) oxide onto 3D spongin scaffolds for effective removal of C.I. Basic Blue 9 dye has also been reported [26]. Moreover, because of its outstanding thermostability (up to 360 °C) and the preservation of its structure during carbonization up to 1200 °C [27], spongin has found application in a novel scientific field described as extreme biomimetics [28,29]. For example, a nanostructured hematite–spongin composite developed using the extreme biomimetics approach has also been recently reported [15]. This biopolymer also shows a high affinity for the extraction of ions of heavy metals such as mercury and copper. In this work, we combined Fe_3_O_4_ nanoparticles and spongin properties into one unique composite, named magnetic spongin, in our attempt to create a sustainable absorbent for organic wastewater treatment.

## 2. Results

### 2.1. XRD Patterns

**Figure 1:**
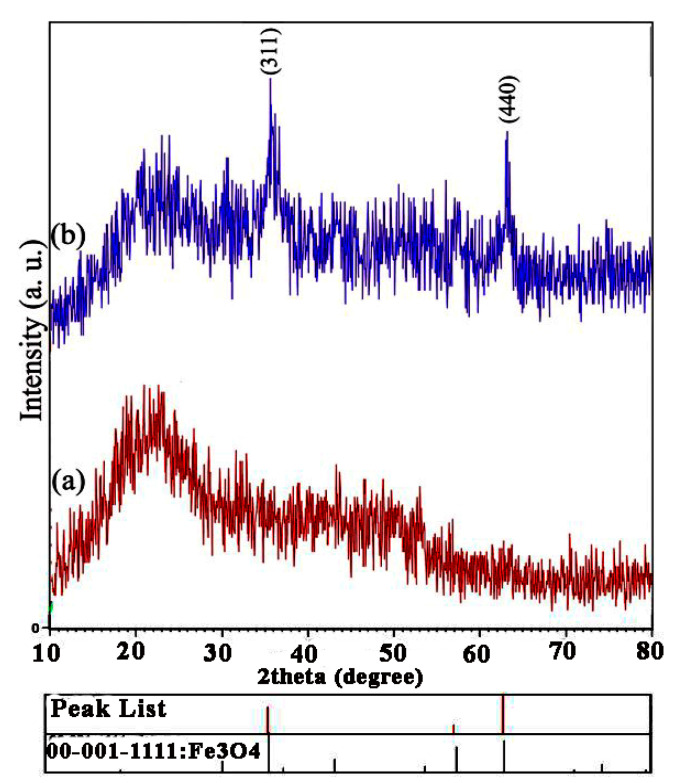
XRD spectra of spongin without iron oxide (**a**) and spongin with iron oxide (**b**).

The XRD patterns of prepared pure spongin (a) and spongin–Fe_3_O_4_ nanocomposites (b) are shown in Figure 1. Figure 1b clearly indicates that for Fe_3_O_4_ nanoparticles, the diffraction peaks appeared at 2Ɵ angles of 30.0, 35.5, 43.1, 53.6, 57.2, and 62.5, corresponding to the (330), (311), (004), (224), (115), and (440) crystal planes, respectively, suggesting a face-centered cubic lattice (fcc) crystal structure (JCPDS card no. 19-0629) [30,31]. This confirms that the preparation procedure did not have an effect on the crystal structure of the ingredients. The strongest peak at 2Ɵ = ~25.5 was attributed to the hkl index (101) [25].

### 2.2. FESEM Images

**Figure 2:**
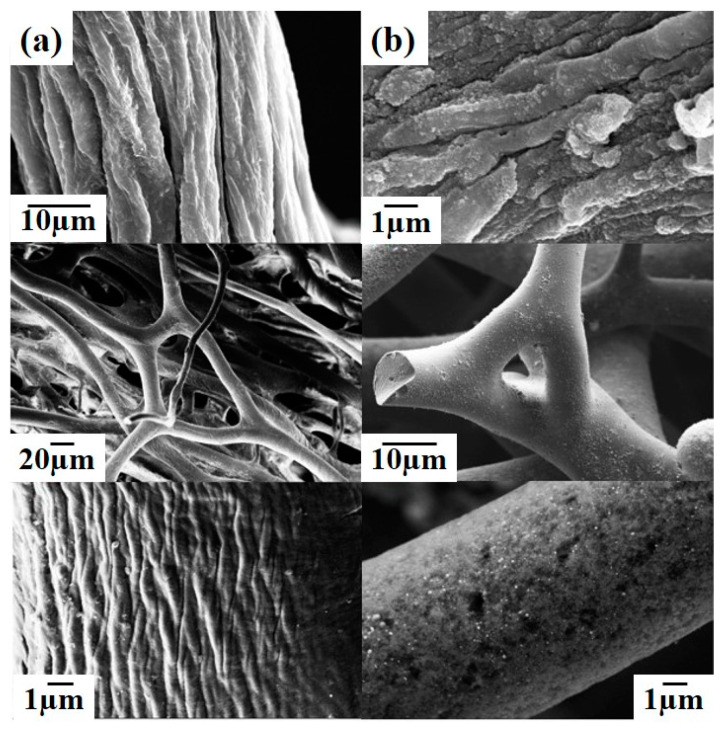
FESEM images of spongin without iron oxide (**a**) and with iron oxide (**b**).

The results of scanning electron microscopy (Figure 2a,b) revealed the surface morphology of the spongin 3D structure prior to and after precipitation of Fe_3_O_4_ particles. Precipitation of Fe_3_O_4_ resulted in the creation of complex foam-like formations by the spongin microfibers, yet the skeleton still had open porosities. The distribution of the nanometric Fe_3_O_4_ particles on the surface of the spongin fibers was clearly visible in the FESEM images, and the washing procedure showed no influence on the location of the obtained Fe_3_O_4_ phase.

### 2.3. VSM Patterns

**Figure 3:**
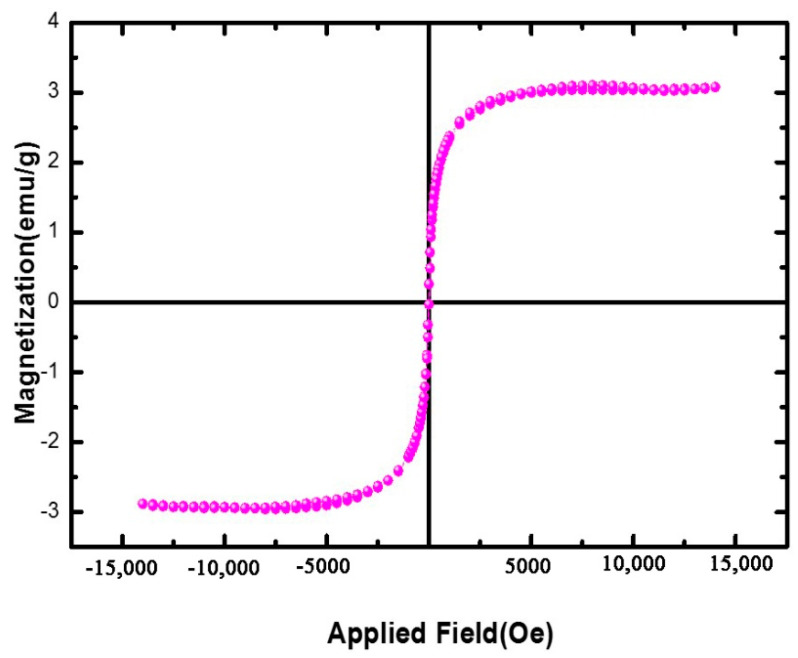
Magnetization curves at room temperature for the studied Fe_3_O_4_-spongin nanocomposites.

Figure 3 shows the results of the VSM analysis of spongin–Fe_3_O_4_. A study of the magnetic spongin–Fe_3_O_4_ nanocomposites revealed that nanoparticles have considerable magnetic properties, with a magnetic saturation value of 3.252 emu/g, which is acceptable for practical applications involving the dispersion of the spongin nanocomposites in water and their collection using a magnetic field [32].

### 2.4. FTIR Spectra

**Figure 4:**
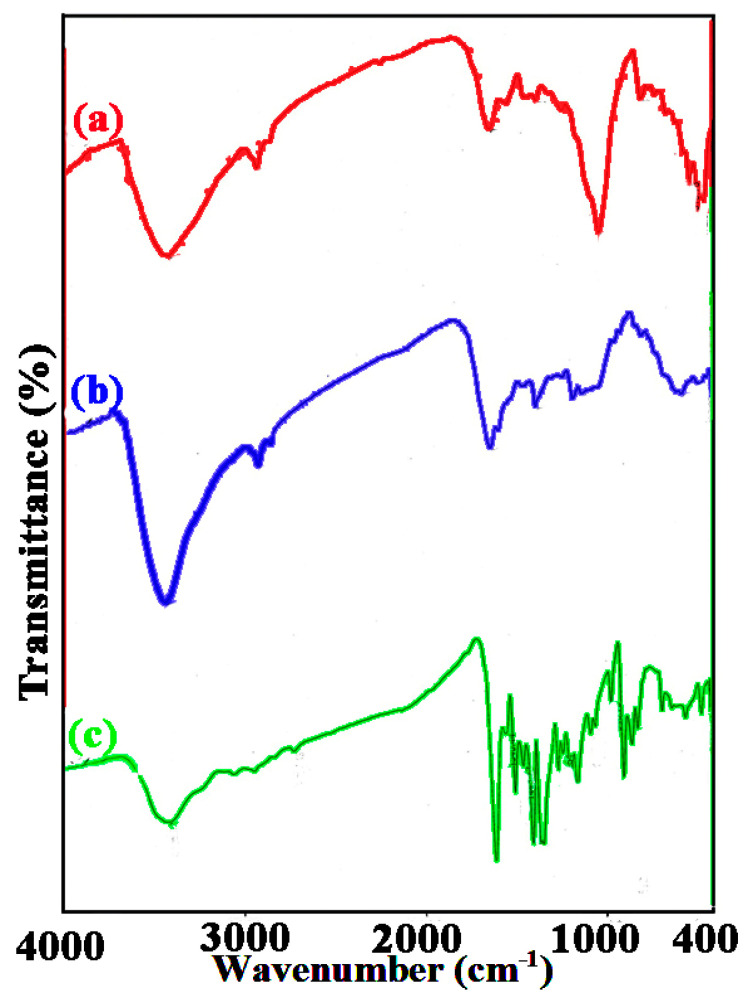
FTIR spectra of (**a**) spongin (**b**) iron oxide-spongin, and (**c**) Fe_3_O_4_-spongin-MB.

FTIR analyses were performed on (a) spongin, (b) spongin–Fe_3_O_4_, and (c) spongin–Fe_3_O_4_–MB samples, and the results are displayed in Figure 4a–c, respectively. In the spongin sample, the wideband extending from 3200 to 3300 cm^−1^ corresponds to the stretching vibrations of the nitrogen–hydrogen bond (of the amide A groups) and O–H groups (Figure 4a) [33,34,35]. Two bands centered at 2923 cm^−1^ are associated with the respective asymmetric and symmetric vibrations of CH_2_ and CH_3_ [36,37].

The stretching vibrations of the N–H and CO groups of amide I (mainly the stretching vibrations of CO) and amide II (stretching of C–H and bending of N–H) cause relatively intense bands at 1630 cm^−1^ and 1520 cm^−1^ [38,39,40]. Furthermore, the amide III band extending from 1220 to 1300 cm^−1^, which can be seen in the case of the spongin samples, is due to the phase combination of carbon–nitrogen bond stretching as well as N–H plane bending vibrations, considerably reducing the peptide bond (–CONH–) footprint in the spectrum of the organic/inorganic material [41,42]. Finally, the N–H band can be observed at 472 cm^−^^1^ (Figure 4a), partially overlapping with the Fe–O band at 563 cm^−1^ (Figure 4b), leading to the lower wavenumbers of the N–H band at 461 cm^−1^ [15,26].

The MB-loaded spongin–Fe_3_O_4_ revealed characteristic changes in the behavior of the hydroxyl and acetyl groups, reflected by the shifts from 3433, 1633 (amide I), and 1560 cm^−1^ (amide II) to 3405, 1699, and 1504 cm^−1^, respectively, after adsorption of MB molecules [43,44,45]. A strong interaction of MB could explain these changes with OH groups and acetyl groups [46]. The presence of all MB sharp bands at 1599 cm^−1^—which corresponds to the C=N and C=C signals—and 1346 cm^−1^—which corresponds to the C‒N signal—verified the adsorption of MB by the spongin–Fe_3_O_4_ composite (Figure 4c) [37].

### 2.5. TGA/DTA

**Figure 5:**
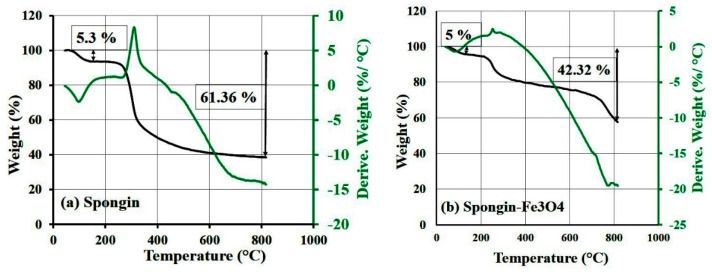
TGA and DTA results of (**a**) spongin and (**b**) Fe_3_O_4_-spongin nanocomposite samples.

TGA was used to determine the Fe_3_O_4_ content of the samples and the thermal stability of the prepared composite. Figure 5a,b present the results of the TGA and DTA curves for the spongin (a) and spongin–Fe_3_O_4_ (b) samples, respectively. The observations indicate that spongin is thermally stable below 300 °C, yet above this temperature, a 50% mass loss is observed due to the melting of the α-chains of spongin (this is similar behavior to that of α-keratin) and dissociation of crosslinks formed through −S−S− and hydrogen bonds or salt bridges [15]. Further increasing the temperature increases the mass loss to 60% due to the decomposition of peptide bonds [26].

The experimental results (Figure 5a,b) first revealed a 5% mass change up to 190 °C, which was attributed to dehydration [47,48]. A further narrow drop in the mass was due to the annihilation of the protein system of the sponge skeleton. In the case of the pure spongin sample, the total mass loss was 61.4%, while for the nanocomposite, this value reached only 42.3% of the total mass. After heating up to 800 °C, the nanocomposite remnants accounted for 23.3% of the total mass, indicating that the spongin–Fe_3_O_4_ nanocomposite contained around 19.0% Fe_3_O_4_ (61.4%−42.3% = 19%). For the spongin–Fe_3_O_4_ nanoparticles, after the evaporation of water molecules at 80–130 °C, a further 21.0% mass loss was observed at 500 °C. We concluded that the spongin–Fe_3_O_4_ adsorbent has higher thermal stability in comparison to pure spongin.

### 2.6. Zeta Potential Measurements

**Figure 6:**
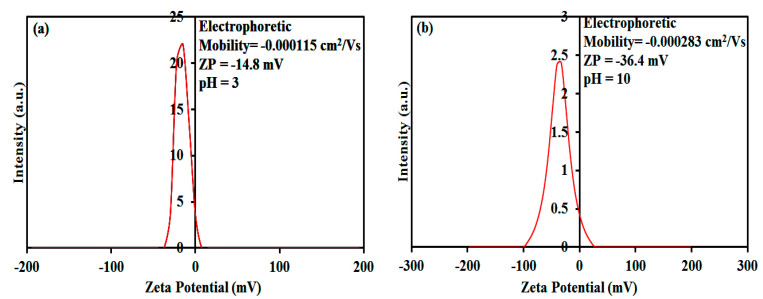
Zeta potential report of Fe_3_O_4_–spongin nanocomposites at (**a**) pH 3 and (**b**) pH 10.

We tested the zeta potential (ZP) of the spongin–Fe_3_O_4_ hybrid at pH 3 and 10 (Figure 6a,b, respectively). ZP analysis of the spongin–Fe_3_O_4_ nanocomposite revealed a negative charge of −14.8 and −36.4 mV at pH 3 and 10, respectively. In this study, the results indicated that cationic MB and CV dyes were loaded into spongin–Fe_3_O_4_ hybrid nanostructures by noncovalent electrostatic interactions. MB and CV are model cationic dyes including –(CH_3_)_2_N^+^ groups, and they firmly exist in solution as positively charged ions within the studied pH range. When pH is between 3 and 10, electrostatic attraction arises between MB or CV (–(CH_3_)_2_N^+^) and the negatively charged (e.g., RS–, RO–, –COO–) functional surface groups –O–H (hydroxyl group), –S–H (thiol group), and -COOH (carboxyl group, amino group (−NH_2_)) of the spongin–Fe_3_O_4_ hybrid [29,49]. 

## 3. Discussion

### 3.1. Investigation of Dye Adsorption

#### 3.1.1. The Effect of Ionic Strength and pH Parameters

**Figure 7:**
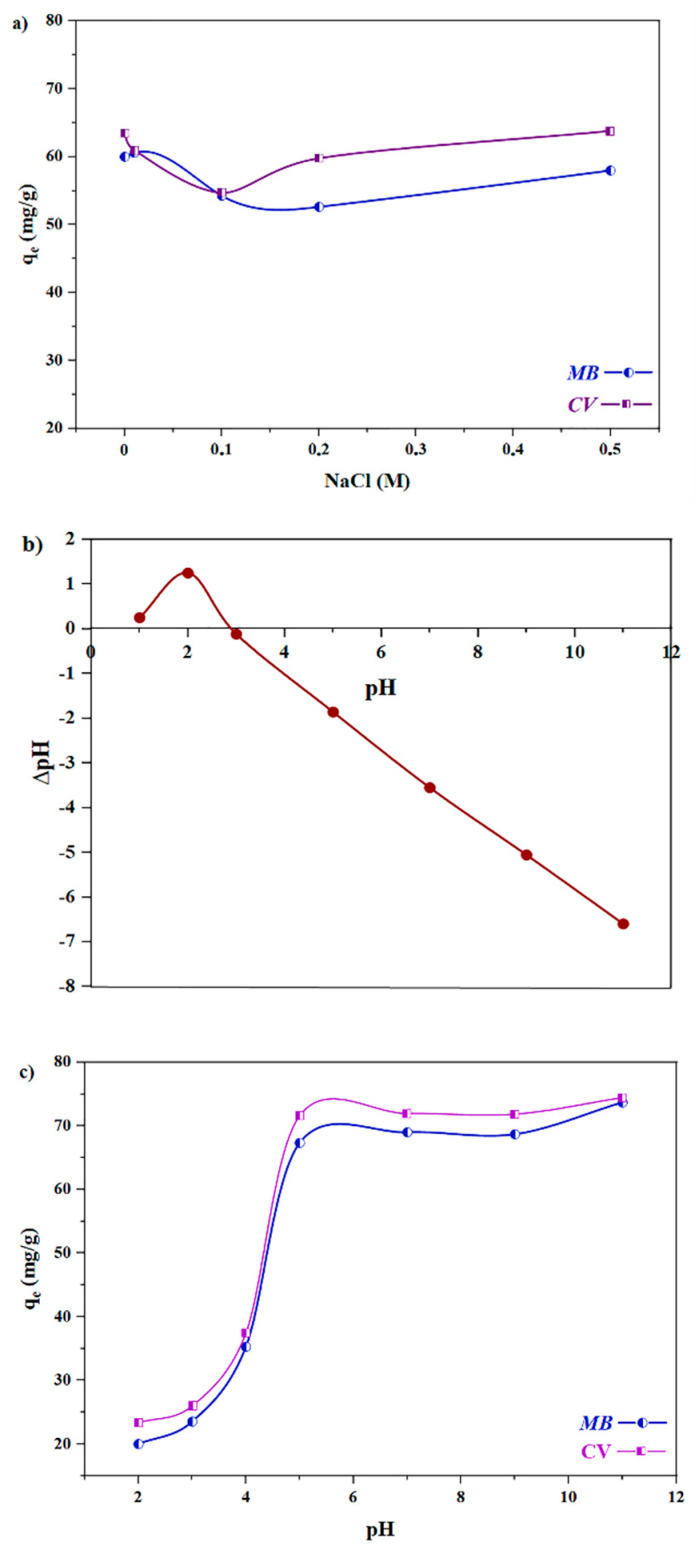
(**a**) The adsorption capacity of the Fe_3_O_4_–spongin nanocomposite under different NaCl concentrations for CV and MB. (**b**) Points of zero charge plot of the Fe_3_O_4_–spongin nanocomposite for determination of pHpzc. (**c**) Influence of the initial pH of the CV and MB solutions on the adsorption capacity of magnetic spongin.

The adsorption capacity of the adsorbents, depending on their nature, can be affected by ionic strength as well as the pH of the dye–wastewater solution. The adsorption of spongin with anionic functional groups (at pH 3–11) is influenced by ion strength via the neutralization of anionic groups by cations. The effect of the ionic strength of the solution containing spongin–Fe_3_O_4_, with spongin as the ingredient with the most active ‒RS–, RO–, and –COO– groups, was tested using NaCl solutions at 0.01–0.5 M concentration ranges. As expected, the result revealed a gradual decrease in the MB adsorption capacity and a slight decrease in the CV adsorption capacity of the magnetic biosorbent with an increase in the NaCl concentration up to 0.2 M (Figure 7a). This reduction can be attributed to the competition between Na^+^ ions and cationic CV and MB in adsorbing onto the negatively charged groups of the bioadsorbents. The mobility of Na^+^ ions is greater than that of MB or CV, because of the significantly smaller size of the Na^+^ (1.02 Å) ions in comparison to MB (15.06 Å) and CV (14.52 Å). Therefore, the surface of the adsorbent is more accessible by the sodium cations than by MB and CV. This could also be due to the shielding of surface groups by Na^+^ ions. Indeed, decreased adsorption capacity due to higher ionic strength caused by the addition of sodium chloride is because of the repulsion between Na^+^ and cationic MB and CV dyes. When the NaCl concentration increases by more than 0.2 M, a slight increase in dye adsorption on the adsorbents is observed. This behavior is caused by the decrease in the solubility of the dyes’ molecules. This reduction due to the increase in the ionic strength of the media can be attributed to the aggregation of dye molecules through physical interaction, including van der Waals forces, ion–dipole forces, and dipole–dipole forces. The adsorption of aggregated dye molecules on adsorbents results in an increase in the dye adsorption capacity of the adsorbent. Yahya et al. reported a similar trend, in that the adsorption of reactive dyes on activated carbon increased alongside the ionic strength of the media [50].

The initial pH of the dye solution has an effect on the surface charge of the adsorbents and the ionization of the dyes, affecting the adsorption process. For evaluation of the effect of this factor, the point of zero charge pH (pHpzc) of the adsorbent should be clarified. To calculate the pHpzc, the related experiment was performed based on the procedure presented in the experimental section (Section 4.6), and the results are presented in Figure 7b. As can be seen in Figure 7b, the pHpzc value of spongin–Fe_3_O_4_ is between 2.6 and 3.0. Therefore, the adsorbent charge at a pH less than the pHpzc is positive, and the adsorbent has the ability to absorb negatively charged species, while at pH levels higher than the pHpzc the adsorbent charge is negative, and has more ability to absorb positively charged species such as cationic dyes. Thus, due to the repulsive forces between the adsorbent and the cationic adsorbate in an acidic environment (pH < pzc), there is a lower adsorption efficiency for cationic dyes in this region. In addition, according to the diagram of the effect of pH on the adsorption of cationic dyes, in an acidic environment, the concentration of H^+^ ions on the adsorbent surface increases, which repels the cationic dye molecules and reduces the adsorption of dye molecules. While in alkaline conditions, the adsorption of cationic dyes also increases with increasing electrostatic gravity. Therefore, the adsorption capacity of the adsorbent was evaluated in the pH 2–11 window.

**Scheme 1:**
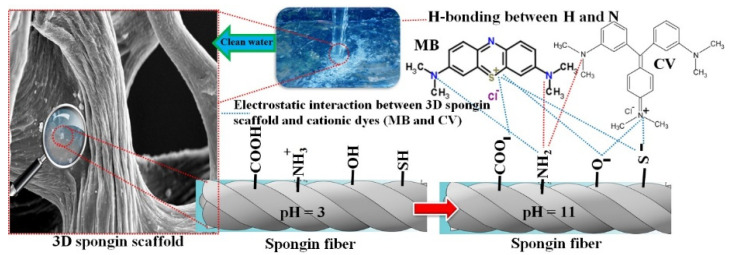
The scheme of electrostatic interaction between the 3D spongin scaffold and the cationic dyes (MB and CV).

As can be seen in Figure 7c, the adsorption of MB and CV revealed a strong sensitivity to pH. Both MB and CV have ammonium groups that are protonated in an extensive pH range. Furthermore, the pH-dependent behavior of spongin is due to the RS-, RO-, and -COO- groups, which become anionic at pH > 3. Figure 7c shows a sharp increase in the adsorption of dyes between pH 3 and 5.5, but further increase in pH has little effect on adsorption. Thus, the dye’s adsorption performance over a wide pH range (pH 5.5–11) is almost identical, and is at the maximum level. Therefore, there is no need to adjust the pH, which is a very good advantage in removing cationic dyes (i.e., CV and MB) from natural waters. This phenomenon can be explained by the increase in the number of anionic groups on the adsorbent, in which the carboxylate groups become negatively charged at a pH of greater than 4.5; thus, the adsorption of cationic dyes increases. Therefore, at pH values between 4.5 and 11.0, the negatively charged spongin scaffold attracts the cationic CV/MB, and the maximum adsorption capacity was observed in this range (see Scheme 1) [51]. On the other hand, the absorption is reversed at pH values below 3, because there is some electrostatic repulsion between the spongin scaffold and cationic dyes. 

#### 3.1.2. Adsorption Kinetics

**Figure 8:**
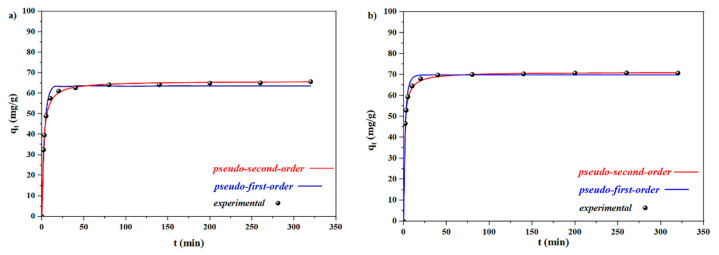
Modeling of the adsorption kinetics of dye removal by Fe_3_O_4_–spongin nanocomposites for (**a**) MB and (**b**) CV, based on two models.

Adsorption kinetics were studied in 300 mg L^−1^ solutions of MB and CV in order to determine the optimal equilibrium time. Figure 8a illustrates the time-resolved adsorption. Obviously, the adsorption of both cationic species is a rapid phenomenon, but the equilibrium states are established after 20 and 10 min, respectively. In the initial stages, adsorption is very fast, thanks to the accessibility of the active sites of the adsorbent. As time passes, this rate decreases, and in the final stage >10 min the adsorbent is saturated. In this work, notwithstanding the decrease in swelling capacity, an increase in dye deletion efficiency was observed. This could be related to the improved anionic centers on Laponite RD structures. The dye adsorption capacity of magnetic spongin for MB was 65.70 mg/g, whereas the adsorption capacities of magnetic spongin–Fe_3_O_4_ were found to be 70.69 mg/g, for CV. 

Pseudo-first-order (Equation (1)) and pseudo-second-order (Equation (2)) kinetic models were tested to determine the rate constant and adsorption capacity of the adsorbent nanocomposites ([52]):

(1) $$q_{t}=q_{e}\left(1-e^{k_{1}t}\right)$$

(2) $$q_{t}=\frac{q_{e}{}^{2}k_{2}t}{1+q_{e}k_{2}t}$$

In these equations, *q_e_* and *q_t_* (mg·g^−1^) represent the amounts of the adsorbed MB and CV species on the adsorbent at the equilibrium conditions, and at any given time (t), respectively. The pseudo-first-order *k*_1_ (min^−1^) and pseudo-second-order *k*_2_ (g·mg^−1^·min^−1^) are rate constants. As can be seen in Table 1 and Figure 8, the data fitted the pseudo-second-order kinetic model better in favor of both MB (0.9921) and CV (0.9910). Moreover, the theoretical adsorption capacity of magnetic spongin–Fe_3_O_4_ for MB and CV calculated according to the pseudo-second-order model (*q_e_*) was found to be 65.97 and 71.07 mg g^−1^, respectively. These results were in good agreement with the experimental values (*q_e.exp_* = 65.70 and 70.69 mg g^−1^ for MB and CV, respectively).

#### 3.1.3. Adsorption Isotherms

The adsorption isotherms presented in Figure 8 are key to designing adsorption systems. One can easily understand that the adsorption capacity values initially grow before reaching a plateau because the concentration is high, the adsorption sites are saturated, and the adsorption stays constant. The Langmuir and Freundlich isotherms were applied using the empirical equilibrium adsorption data.

The Langmuir model is valid for monolayer adsorption phenomena, and assumes that the adsorption occurs at particular homogeneous positions throughout the adsorbent. This model is explained by Equation (3) [53]:

(3) $$q_{e}=\frac{q_{m}K_{L}C_{e}}{1+K_{L}C_{e}}$$

In this formula, *K_L_* represents the Langmuir adsorption constant (L/mg), *q_m_* is the maximum theoretical capacity (mg/g), and *C_e_* is the equilibrium concentration of the dye molecules in the solution (mg/L). The dimensionless adsorption constant *R_L_*, is further given by:

(4) $$R_{L}=\frac{1}{1+K_{L}C_{0}}$$

where *C*_0_ is the initial concentration of methyl blue dye and *K_L_* is the Langmuir constant (L/mg). The nature of the isotherm is defined by the magnitude of *R_L_*, which is a key value in the Langmuir model (if unfavorable, *R_L_* is greater than 1; if adsorption is linear, *R_L_* will be equal to 1; the phenomenon is favorable if *R_L_* is between 0 and 1, and it is irreversible if *R_L_* = 0 ([50]). The Freundlich model, on the other hand, assumes that the adsorption takes place on a heterogeneous, multilayered surface, and that the capacity increases with the concentration of the adsorbate. This model is expressed as follows ([54,55];):

(5) qe=kfCe1n

where *n* is a constant indicating adsorption intensity, and *k_f_* is the equilibrium adsorption coefficient (mg ^(1−n)^ L^n^ g^−1^), which is the Freundlich constant expressing the adsorption capacity.

**Figure 9:**
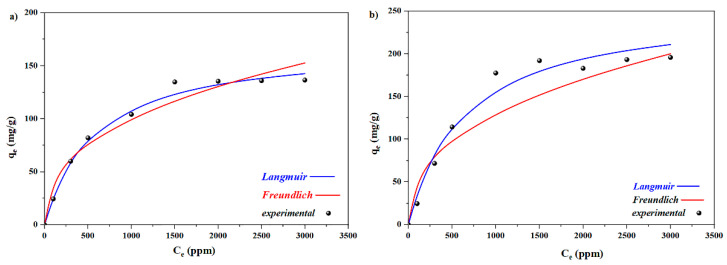
Modeling of adsorption isotherms by implementing the Freundlich and Langmuir models to remove (**a**) MB and (**b**) CV with Fe_3_O_4_–spongin nanocomposite).

The Langmuir and Freundlich isotherms of the two cationic dyes can be seen in Figure 9, and all corresponding factors are summarized in Table 2. The large correlation coefficient (*r*^2^ > 0.97) in the case of the Langmuir equation indicates that the adsorption of the dyes on m-spongin is Langmuir monolayer adsorption. The *R_L_* values of the Langmuir model ranged from 0 and 1, further reflecting the favorability of the adsorption. This finding shows that the magnetic hydrogels exhibit encouraging efficacy in the adsorption of MB and CV from aqueous solutions. Increasing the amount of spongin–Fe_3_O_4_ enhanced the Langmuir *K_L_*, reflecting the high tendency of the adsorbent spongin–Fe_3_O_4_ to adsorb the dye molecules ([56]).

If the Freundlich n values range from 0 to 10, the adsorption is feasible [57]. The maximum adsorption capacities (*q_max_*) for MB and CV were 168 and 253 mg/g, respectively. This is comparable to the values obtained with wheat shells (21.5 mg/g), leaves of spongin trees (19.9 mg/g), date pit carbon (455 mg/g), and Fe_3_O_4_ nanocomposites with 3D spongin (216.3 mg/g) ([58,59,60,61]).

#### 3.1.4. Thermodynamic Studies

The relationship between the adsorption of the dyes and temperature was monitored at 273, 298, and 318 K using 300 mg/L solutions of the target species, and thermodynamic factors such as Δ*H*, Δ*S*, and Δ*G* were determined using the following equations ([62]):

(6) $$K_{c}=\frac{C_{s}}{C_{e}}$$

(7) $$LnK_{c}=-\frac{\Delta H}{RT}+\frac{\Delta S}{R}$$

(8) ΔG=ΔH−TΔS

In these equations, *C_s_* and *C_e_*, are the concentrations of each dye in the adsorbent and solution phases, respectively, while *K_c_* is the equilibrium constant, *R* is the universal gas constant (8.314 J mol K^−1^), and *T* is the absolute temperature in Kelvin (K).

**Figure 10:**
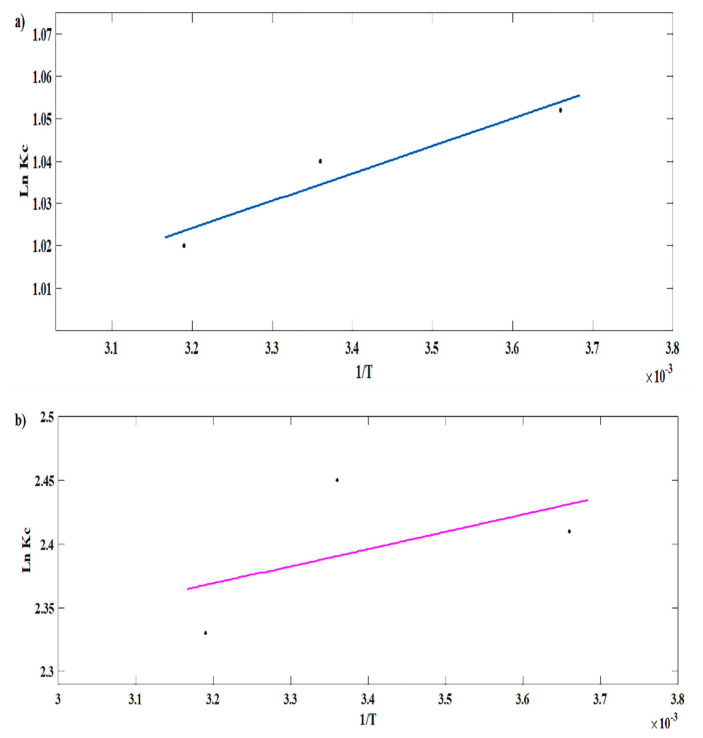
The calculation of thermodynamic factors for (**a**) MB and (**b**) CV adsorption on the magnetic spongin.

The slope and intercept of the plot of *Ln*(*K_c_*) against *1*/*T* (Figure 10a,b) are Δ*H* and Δ*S*. The calculated data are summarized in Table 3. According to the obtained data, endothermic specificity was indicated for the process of MB adsorption on spongin due to the positive value of Δ*H*; at the same time, it was exothermic for CV due to the negative value of Δ*H*. However, Δ*G* for the adsorption processes of both MB and CV on the 3D spongin scaffold was negative, which explains their feasibility and spontaneous natures. Moreover, the increase in the negative value of Δ*G* with increasing temperature revealed the dependency of adsorption on temperature, with an increasing efficiency of adsorption processes at higher temperatures. The Δ*S* values were positive for both MB and CV adsorption processes, suggesting increased disorder at the adsorbent–solution interface.

### 3.2. Desorption Studies

**Figure 11:**
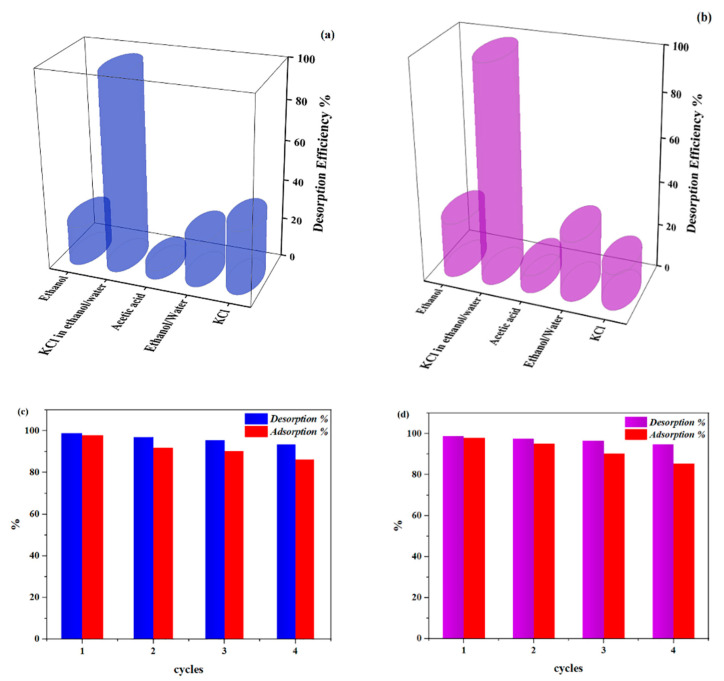
Desorption efficiency of (**a**) MB and (**b**) CV from magnetic spongin by various solutions. Adsorption–desorption of (**c**) MB and (**d**) CV from magnetic spongin using 0.1 M KCl (ethanol: H_2_O 50:50) for 4 cycles.

Adsorption capacity is a key factor in choosing the proper adsorbents, yet recyclability is also very important from an economic perspective. In order to determine this factor, desorption experiments were performed using ethanol, ethanol/water (50:50, V:V), acetic acid 0.2 M, KCl 0.05 M, and KCl 0.5 M in water/ethanol (V:V, 50:50) solutions, the results of which can be seen in Figure 11a–d. Based on our observations, a solution of KCl in water/ethanol led to the best regeneration of the used adsorbent for both MB and CV (96% and 98%, respectively). These data are consistent with the effect of ionic strength described above. The experiment reveled good desorption behavior for dyes in both KCl and ethanol solutions, but enhancing ethanol solution to KCl solution increased the desorption of the dyes, which is remarkable. Tests using acetic did not show the expected results (only around 2% for both dyes), but this could be explained by the pH dependence of the adsorbent capacity described above.

Recyclability tests indicated that after four adsorption/desorption cycles, a small decrease of ~6% in the adsorption capacity of the adsorbent for both dyes takes place, indicating the considerable promise of the magnetic sponge adsorbent for repetitive applications.

Using FTIR spectra, pH studies, ionic strength assessments, and adsorption–desorption studies, the adsorption mechanism was determined. Based on the observations, both weak interactions and electrostatic interactions of the hydroxyl, carboxylic, amino, and thiol groups are the reasons for the effective behavior of the adsorbent (Table 4). The presence of hydrogen bonds and electrostatic interactions was verified by the characteristic shifts of the ‒OH group’s adsorption in the FTIR spectra. The pH insensitivity of the adsorption of 3D spongin–Fe_3_O_4_ indicates that the adsorption is dependent on the carboxylic groups, which adopt an anionic form at pH > 3. Additionally, the decreased adsorption capacity at higher ionic strengths supports the electrostatic nature of the interactions.

The functional properties of magnetic spongin for the adsorption of MB and CV dyes have been compared with some previously published works (Table 4). As shown in the table, magnetic spongin exhibits good performance, and its adsorption properties are comparable to most other adsorbents, while its method of synthesis is very simple, cheap, and renewable. In addition, magnetic spongin shows a suitable adsorption response in a wide pH range (5.5–11), which is a great advantage.

## 4. Materials and Methods

### 4.1. Materials

Analytical-grade FeCl_2_.6H_2_O and FeCl_3_.6H_2_O were used as received from Merck. Methylene blue (Mw = 319.85 g mol^−1^, molecular formula C_16_H_18_ClN_3_S, and λ_max_ = 664 nm) was supplied by Alvan Co., Iran (Tehran, Iran). A Smart-2-Pure, TKACo ultrapure water instrument was used for preparing deionized water. Crystal violet dye (dye content ≥ 90%, CI = 42,555, M_W_ = 407.99 g mol^−1^ as well as λ_max_ = 590 nm, molecular formula C_25_H_30_ClN_3_) was procured from Merck Co. (Darmstadt, Germany).

### 4.2. Preparation of Spongin Scaffolds

Spongin scaffolds were extracted from *Hippospongia communis* demosponges via 180 min of washing with deionized water to remove cells, microorganisms, and residual salts [26], followed by soaking in a 0.5% NaOH solution for 60 min at 37 °C. The treated material was then frequently rinsed with deionized water, and subsequently placed in a 5% KMnO_4_ bath for 15 min for bleaching under ambient conditions, followed by repeated rinsing with deionized water until the pH of the washing water reached 6.5. The bleached sample was eventually positioned in a 3 M solution of hydrochloric acid at room temperature for three days to dissolve possible remaining calcium carbonate microparticles originating from the shells of mollusks or crustaceans. During this period, the HCl solution was renewed every 12 h. Finally, the produced sponge material was cleaned using distilled water, dried in air, and placed in a plastic box at room temperature for one day.

### 4.3. Preparation of Magnetic Spongin–Fe_3_O_4_

The spongin–Fe_3_O_4_ was prepared through precipitation of Fe^3+^ and Fe^2+^ in a mixture containing a piece of spongin. Typically, a 2 × 2 cm piece of spongin was placed in 200 mL of deionized water under sonication at 50 kHz for 30 min at 70 °C. This was followed by dissolving a 2:1 mixture of Fe^3+^/Fe^2+^ (i.e., 3 g of FeCl_3_·6H_2_O and 1.54 g of FeSO_4_·7H_2_O) in 5 mL of water. This blend was then added to the spongin mixture under stirring, and the resulting mixture was stirred for a further 10 min. Then, a 3 M NH_3_ solution was added to the mixture to obtain the final product by increasing the solution’s pH to 10 at 70 °C. The resulting dark solution was stirred for a further 60 min. The obtained magnetic spongin nanoparticles were then cleaned with water until the pH of the washing water reached 7.0.

### 4.4. Characterization of the Synthesized Spongin–Fe_3_O_4_ Nanocomposite

An LEO-1455VP SEM instrument was used to perform SEM analyses (Carl Zeiss, Oberkochen, Germany) and EDX analyses (Carl Zeiss, Oberkochen, Germany). The voltage was increased to up to 20 kV in order to examine the composition and crystal properties of the adsorbent. The size of the coherent scattering region of the particles was calculated using empirical data and the Debye–Scherrer equation [70]:

D_c_ = Kλ/βcosθ (9)

where D represents the particle diameter, β expresses the diffraction peak width at half maximum, θ shows the diffraction angle for the peak, and λ is 0.1540 nm.

Room temperature magnetic analyses were performed using a vibrating sample magnetometer (VSM, Meghnatis Daghigh Kavir, Kashan, Iran). UV–Vis diffuse reflectance spectra were attained using a Shimadzu UV-2550 instrument (Shimadzu, Tokyo, Japan). The FTIR spectra were collected between 400 cm^−1^ and 4000 cm^−1^ with a Nicolet-Impact 400D instrument and KBr bromide pellets. An SZ-100z Dynamic Light Scattering and Zeta Potential Analyzer (Horiba Jobin Yvon, Horiba, Kyoto, Japan ) was used to measure the zeta potential of the prepared nanocomposite. 

### 4.5. Pollutant Adsorption Tests

The study of adsorption of the dyes was carried out in batch mode. The tests involved adding 20 mg of the adsorbent to 25 mL of a 300 mg/L solution of MB and CV and shaking at 120 rpm. For the kinetic data collection, 0.5 mL aliquots of the test solutions were examined at known times before and after the onset of the reactions, and their dye concentrations were ascertained from their UV–Vis spectra at 590 and 664 nm for CV and MB using a Shimadzu UV-1800 instrument. The adsorption capacity values ($q_{t}$) were calculated using the following expression:

(10) $$q_{t}=\frac{\left(C_{0}-C_{t}\right)}{m}\times V$$

where *C*_0_ and *C_t_* are the starting and final concentrations, respectively of MB and CV at time *t*, while *m* (g) and *V* (L), expressing the quantity and volume, respectively, of the adsorbent and the test solution.

Using 0.1 M HCl and sodium hydroxide to alter the sample solutions’ pH, the effect of pH on the adsorption efficiency was studied. Similar studies were performed on the effect of ionic strength by adding various amounts of NaCl to the test solutions after an aging period of one day to ensure equilibrium. The thermodynamic factors corresponding to the process were determined via experiments using a thermostatic shaker at 0 °C, 25 °C, and 45 °C for one day. Isothermal tests were conducted in a thermostatic shaker at 25 °C for one day to ensure equilibrium, while the concentrations of MB and CV varied in the range of 100–3000 mg/L.

### 4.6. Points of Zero Charge

The points of zero charge (pH_pzc_) of adsorbents were determined according to the literature [71,72]. In this series of experiments, 20 mL of 0.05 M NaCl solution was poured into several beakers, and the pH of NaCl solutions was adjusted from 1 to 12 by adding either 0.1 M HCl or 0.1 M NaOH solutions. After setting the pH, the volume of the solution was adjusted to 30 mL by adding 0.05 M NaCl solution. The initial pH (pHi) of the solutions was recorded; then, 50 mg of adsorbent was immersed into solutions and shaken with a shaker (90 rpm, ambient temperature) for 48 h. Then, the solutions containing adsorbents were centrifuged (4500 rpm) for 15 min, and the pH of the supernatant solution was recorded (pH_f_). The points of zero charge were found from the plot of ∆pH (pH_i_-pH_f_) versus pH_i_.

## 5. Conclusions

In situ precipitation of iron salts in a dispersion of 3D spongin scaffold fragments was used to prepare a spongin-based construct with attached magnetic Fe_3_O_4_ nanoparticles for the first time. The results reflected the promising properties of the magnetic spongin–Fe_3_O_4_ composite in the removal of selected cationic dyes (i.e., CV and MB) from simulated wastewater. The magnetic 3D spongin–Fe_3_O_4_ showed considerable adsorption efficiency. This sorbent had other benefits, including ease of synthesis, recovery, lack of secondary pollutants, low cost and, most importantly, eco-friendliness. Removal of organic dyes and other contaminants is essential to ensure healthy water and prevent various diseases. On the other hand, in many cases, dyes are used as models to demonstrate the adsorption properties of nanostructures. Due to the good absorption properties of magnetic spongin, it can be proposed as a green and uncomplicated carrier in drug delivery applications.

## Figures and Tables

**Table 1:** Calculated kinetic parameters of kinetic models for the removal of CV and MB.

	Pseudo-First-Order			Pseudo-Second-Order			
	*k*_1_ (min^−1^)	*q_e_* (mg·g^−1^)	*r* ^2^	*k*_2_ × 10^3^ (g·min^−1^·mg^−1^)	*q_e_* (mg·g^−1^)	*r* ^2^	*q_e.exp_* (mg·g^−1^)
**MB**	0.3189	63.55	0.9725	7.993	65.97	0.9921	65.70
**CV**	0.4865	69.86	0.9551	13.7	71.07	0.9910	70.69

**Table 2:** Estimated isotherm parameters based on the Freundlich and Langmuir models to remove CV and MB.

	Freundlich			Langmuir			
	*n*	*k_f_* (mg·g^−1^)(g·L^−1^)^−1/n^	*r* ^2^	*q_m_* (mg·g^−1^)	*K_L_* (L·g^−1^)	*r* ^2^	*R_L_* (mg·g^−1^)
**MB**	2.608	7.091	0.9149	168	0.001876	0.9775	0.9815
**CV**	2.545	8.609	0.8674	253	0.001671	0.9642	0.9966

**Table 3:** Calculated thermodynamic parameters for the removal of CV and MB by spongin at various temperatures.

	*T* (K)	Δ*G* (J·mol^−1^)	Δ*H* (J·mol^−1^)	Δ*S* (J·K^−1^·mol^−1^)
**MB**	273	−2392.7 ± 42.1	−539.3 ± 42.1	6.789 ± 0.8
	298	−2562.42 ± 42.1		
	313	−2664.26 ± 42.1		
**CV**	273	−5520.3 ± 120.8	−1125 ± 120.8	16.1 ± 2.1
	298	−5922.8 ± 120.8		
	313	−6164.3 ± 120.8		

**Table 4:** Different adsorbents used to remove the MB and CV dyes.

Dye	Adsorbent	Adsorption Capacity (mg.g^−1^)	Reference
**MB**	Bamboo-based activated carbon	454	[63]
**MB**	Magnetic Fucus vesiculosus	577.1	[63]
**MB**	Coconut shell activated carbon	277	[63]
**MB**	Rice husk activated carbon	343	[63]
**MB**	Magnetic polyvinyl alcohol/Laponite RD	251	[64]
**MB**	Jute fiber carbon	225	[65]
**MB**	Magnetic spongin	168	Present work
**CV**	OC-BzM nanoparticles	248	[66]
**CV**	Magnetic Fucus vesiculosus	1062	[63]
**CV**	CarAlg/MMt nanocomposite hydrogel	88	[67]
**CV**	Nanomagnetic iron oxide	16	[68]
**CV**	Magnetically modified activated carbon	67	[68]
**CV**	Gum arabic-cl-poly(acrylamide) nanohydrogel	99	[69]
**CV**	Magnetic 3D spongin–Fe3O4	253	Present work
